# Differentiation of mtDNA Methylation in Tissues of Ridgetail White Prawn, *Exopalaemon carinicauda*

**DOI:** 10.3390/ani15142037

**Published:** 2025-07-11

**Authors:** Muchen Jiang, Jiayi Gao, Xinyu Zhou, Hao Zhong, Sichen Zhang, Jing Xu, Fei Yu, Xiaofang Lai, Binlun Yan, Huan Gao

**Affiliations:** 1 Jiangsu Key Laboratory of Marine Biotechnology, Jiangsu Key Laboratory of Marine Resources and Environment, Jiangsu Ocean University, Lianyungang 222005, China; 13952212299@163.com (M.J.); 15645132827@163.com (J.G.); ako0404@163.com (X.Z.); 18068785108@163.com (H.Z.); sichenzhang1999@163.com (S.Z.); sweetcabin@163.com (J.X.); lai.xiaofang@163.com (X.L.); yanbinlun1962@163.com (B.Y.); 2 Jiangsu Provincial Collaborative Innovation Center for Marine Biological Industry Technology, Lianyungang 222005, China; 3 Jiangsu Provincial Platform for Conservation and Utilization of Agricultural Germplasm Resources, Nanjing 210014, China; 4 Lianyungang Marine and Fishery Development Promotion Center, Lianyungang 222005, China; yufei6@126.com

**Keywords:** Mitochondria genome, DNA methylation, BSP sequencing, DNA methyltransferases, *Exopalaemon carinicauda*

## Abstract

In a previous study, we found that mitochondrial DNA methylation occurred in the muscle tissue of *Exopalaemon carinicauda* under starvation stress. To explore whether this phenomenon also existed in other tissues, we used the bisulfite method (BSP) to detect the methylation of the mitochondrial genome in the intestinal tissues, hepatopancreas, gills, eye stalks, muscles, heart, and other tissues before and after starvation. In situ hybridization and qPCR techniques were used to analyze the expression of DNMT1 and DNMT3b involved in methylation regulation in different tissues. The results showed that the methylation rate was highest in intestinal tissue, followed by hepatopancreas, gills, heart, muscle, and eye stalk. Significantly different expression levels of DNMT1 and DNMT3b were found in the intestine and hepatopancreas with a higher expression pre-starvation and a lower expression post starvation. The expression levels of DNMT1 and DNMT3b in heart and muscle increased after starvation. The expression levels of DNMT1 and DNMT3b in the eye stalk were low and decreased significantly after starvation. The in situ hybridization of DNMT1 and DNMT3b further verified the results: the mRNA signal in intestinal and hepatopancreatic tissues of the starvation group was significantly weaker than that of the control group. No significant difference in mRNA signal intensity was found in the gill, muscle, and heart tissues of the starvation group compared with the control group. The mRNA signal in the eye stalk tissue of the starvation group was weaker than that of the control group. This study is the first to confirm different levels of mtDNA methylation in different tissues of *E. carinicauda*, which may be closely related to their biological functions.

## Simple Summary

Mitochondrial DNA methylation affects the level of energy metabolism in organisms. In a previous study, we found that mitochondrial DNA methylation occurs in muscle tissues of the ridgetail white prawn (*Exopalaemon carinicauda*) under starvation stress, but it is not clear whether this phenomenon exists in other tissues. Therefore, in this study, we investigated the presence of methylation in other tissues of the ridgetail white prawn, under starvation stress, and whether different levels of methylation existed in different tissues. The results showed that methylation was present in all tissues and at different levels, which may be related to the biological functions of the tissues. This study is the first to suggest that different mitochondrial DNA methylation levels occur in different tissues of crustaceans, which provides a theoretical basis for the study of other organisms and a genetic strategy for the effective selection and breeding of crustacean species.

## 1. Introduction

DNA methylation, an important modification mode in epigenetics, involves the transfer of methyl groups to specific bases like cytosine. The process is catalyzed by methyltransferases (DNMTs) using the active methyl compound S-adenosylmethionine (SAM) as a methyl donor [1,2,3]. The DNA methyltransferase family (DNMTs) is classified into DNMT1, DNMT2, and DNMT3 [4,5,6]. DNMT1 is primarily responsible for the maintenance of DNA methylation modifications [7,8,9,10]. DNMT2 is also classified as a DNA methyltransferase due to its high similarity to DNMT1 and its weak DNA methylation catalytic activity [11,12,13,14]. DNMT3 is a de novo methyltransferase, responsible for the reestablishment of DNA methylation, and it is mainly expressed in undifferentiated cells and during embryonic development [15,16,17].

Mitochondria are known as the “energy factory of the cell”, and they not only directly provide energy for the life activities of the body but also participate in the metabolism of the programmed death of cells, the production of reactive oxygen radicals, and the transport of calcium [18]. Researchers have confirmed the presence of DNMT-catalyzed DNA methylation modifications in the mitochondrial genome [19,20,21,22]. Coincidentally, our previous study found that the COX3 and ND2 gene sequence regions in the mitochondrial genome of muscle tissue were methylated in the ridgetail white prawn, *Exopalaemon carinicauda,* under starvation stress. Because the mitochondria are responsible for energy metabolism, and while the electron transport chain for energy transfer within mitochondria is relatively understood, it is not clear whether and how mitochondrial DNA itself is involved in energy metabolism. Since starvation, an external environmental factor, is directly related to shrimp energy supply, we wanted to explore how the mitochondrial genome is involved in regulating energy metabolism by altering starvation as an external factor. In this study, the bisulfite sequencing PCR (BSP) technique was used to analyze the DNA methylation levels of different tissues of *E. carinicauda*. Simultaneously, the expression of DNMT1 and DNMT3b and the spatial distribution of their signals were further studied using qRT-PCR and in situ hybridization technology to reveal the relationship between methylation and the biological functions of tissues.

## 2. Materials and Methods

### 2.1. Ridgetail White Prawn, Exopalaemon carinicauda

The ridgetail white prawn, *Exopalaemon carinicauda*, is a lower invertebrate, and our experiments on this prawn do not involve animal ethics. To minimize any differences in genetic background, adults were selected from the same family that was self-bred in the laboratory. After 10 days of starvation treatment, six tissues (eye, heart, gill, intestine, muscle, and hepatopancreas) were collected from 100 adult shrimps. Among them, 30 individuals from each of the control and starvation groups were used to analyze the methylation rate of mtDNA in different tissues. Nine individuals from each of the control and starvation groups were used to analyze the expression levels of DNMT1 and DNMT3b in each tissue. Finally, two individuals from each of the control and starvation groups were used to analyze the localization of mRNA signals in each tissue.

### 2.2. Experimental Methods 

#### 2.2.1. DNA Extraction and Methylation 

**Figure 1:**
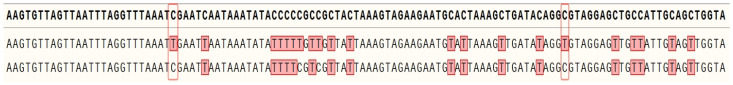
mtDNA methylation site comparison. Note: From top to bottom, 21# methylation sites, unmethylated sequences, and methylated sequences.

**Figure 2:**
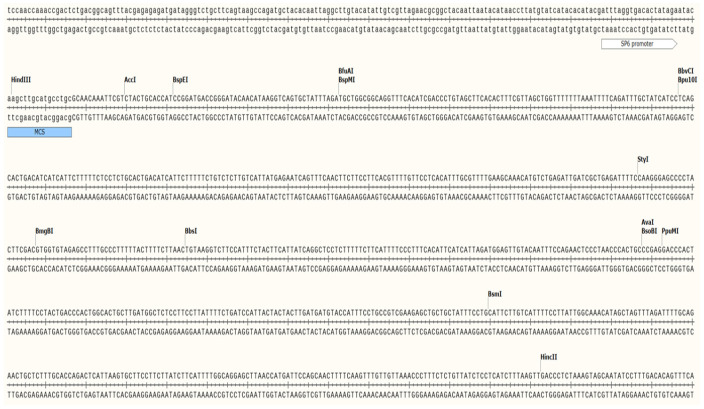
Probe amplification sequence of DNMT1.

**Figure 3:**
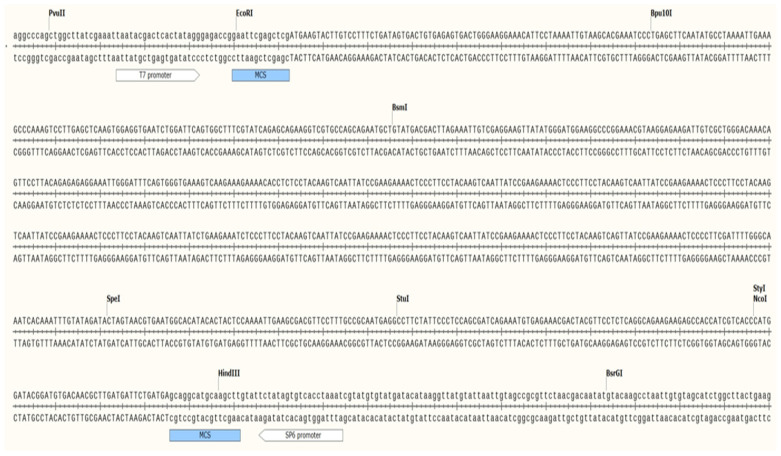
Probe amplification sequence of DNMT3b.

Genomic DNA was extracted from six tissue types and subjected to bisulfite con-version. PCR amplification was performed using mtDNA methylation-specific BSP primers and BSP-amplified primary sequences (No. 16 and No. 21, Table 1; 16# and 21#, Table 1) previously developed by our research group for *E. carinicauda* mitochondrial genome analysis [23]. The primer sequences are shown in Table 2. The PCR protocol consisted of initial denaturation at 95 °C for 2 min, followed by 30 cycles of denaturation (95 °C, 30 s), annealing (55 °C, 30 s), and extension (72 °C, 45 s). The final products were stored at 4 °C. Following the gel extraction of the PCR products, sequencing was conducted by Sangon Biotech (Shanghai, China). Tissue-specific methylation rates were calculated by aligning the sequencing results with the reference mitochondrial genome of *E. carinicauda* and the analysis of the bisulfite sequencing data. Examples of specific comparisons are shown in Figure 1, Figure 2 and Figure 3. The methylation in the amplified region of each primer was mainly CpG methylation, while CHG and CHH types of methylation were also present. The methylation rate (%) was calculated as follows:

Methylation rate (%) = (Amount of methylated sites/Total amount of sites) × 100%

#### 2.2.2. Extraction of Total RNA and Synthesis of First Strand of cDNA

A Trizol Total RNA extraction kit (Shangon, Shanghai, China) was used to extract RNA from the samples. According to the instructions of the HiScript II Q RT SuperMix for qPCR (Vazyme, Nanjing, China), the extracted RNA was reversely transcribed into cDNA and diluted to 50 ng/μL for subsequent experiments.

#### 2.2.3. Analysis of DNMT1 and DNMT3b Expression Characteristics

The qRT-PCR primers used for gene expression analysis are shown in Table 1. 18s rRNAs were used as the internal reference gene and six tissues as a template, and the fluorescence quantification analysis was performed according to the instructions for the ChamQ SYBR qPCR Master Mix (Vazyme, Nanjing, China), using the 2^−ΔΔCT^ method to analyze the quantitative results and SPSS 26.0 software to conduct the one-way ANOVA (*p* < 0.05 indicated a significant difference).

#### 2.2.4. In Situ Hybridization

The recombinant primers were designed according to the core sequence of the *DNMT1* and *DNMT3b* genes. The linearized DNA was amplified with linearized primers and DNMT1-PSPTl8 and DNMT3b-PSPTl8 recombinant plasmids. The primers are shown in Table 1. The DIG RNA labeling kit (SP6) (Vazyme, Nanjing, China) reagent was used for the in vitro transcription. After the DIG-labeled probes were synthesized, the integrity of the combined probes was detected by 1% agarose gel electrophoresis. The probe concentration was determined by a nucleic acid analyzer and stored at −80 °C for use later.

Six tissues of *E. carinicauda* were fixed in 4% paraformaldehyde for 24 h and then routinely dehydrated, embedded in paraffin, and sectioned (thickness 4 μm). The syn-thetic mRNA probe of the *DNMT1* and *DNMT3b* genes was used for pre-hybridization and hybridization experiments, and the DAB chromogenic agent was used for color development. After dehydration in an ascending ethanol gradient and then the use of xylene, neutral gum was used as a sealant. A microscope was used to observe and take micrographs, and the hybridization signal was determined.

The mRNA signals were analyzed for differences in expression by colorimetric comparisons via Photoshop software 2024.

#### 2.2.5. Statistical Analysis

All data are presented as mean ± SEM. The mitochondrial genomes of the control and starvation groups of ridge tail white prawn were amplified using primers 16# and 21#, and the amplified products were sequenced and then aligned with the original sequences using Snapgene 6.0.2 to confirm the methylation profiles. The methylation rates were then calculated. Datasets were evaluated for the homogeneity of variance (Levene’s test) and for normality (the Shapiro–Wilk test). For the data to meet the parametric assumptions, a one-way ANOVA was performed with treatment (pre-starvation vs. post-starvation) as independent factors, followed by Bonferroni post hoc tests for multiple comparisons. The six tissues were analyzed separately. All analyses were performed with SPSS 26.0, with *p* < 0.05 considered statistically significant.

## 3. Results 

### 3.1. Methylation Levels of Mitochondrial Genomes in Different Tissues 

After 10 days of starvation stressing, the statistical results for the mitochondrial genome methylation rate in each tissue of individuals in the starvation and control groups were determined (Table 3). After detecting mtDNA methylation in different tissues of the control group, we did not find any methylation sites. The number of 16&21 methylation sites in the intestine was 206, and the methylation rate was 36.29 ± 2.16%, which was significantly higher compared to several other tissues. The next highest tissue was the hepatopancreas, with 184 methylated sites and a methylation rate of 30.5 ± 1.46%. Next, the gills had 151 methylation sites and a methylation rate of 26.4 ± 0.98%. The number of methylated sites was 124 and 109 for the heart and muscle, respectively, and the methylation rates were 21.9 ± 0.83 and 19.3 ± 0.76, respectively, which were statistically similar. The number of methylation sites in the eye stalk was 31, and the methylation rate was 5.76 ± 0.78, which was much lower compared to other tissues.

### 3.2. Expression Characteristics of DNMTs in Different Tissues 

**Figure 4:**
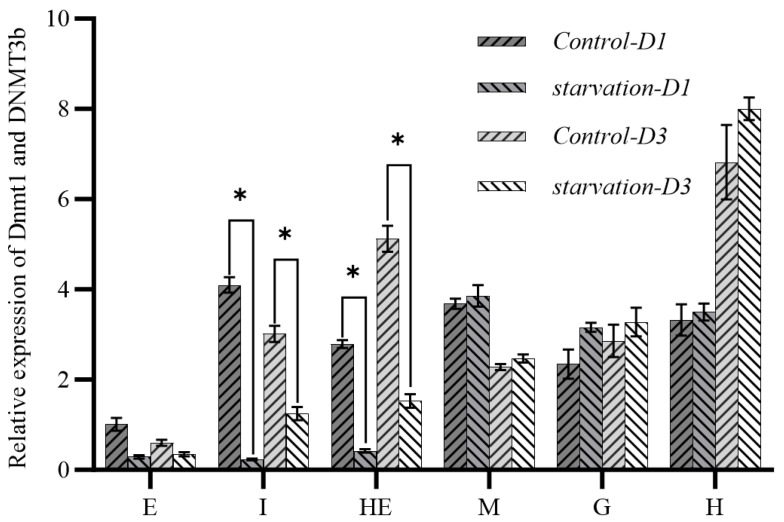
Expression characteristics of *DNMT1* and *DNMT3b* genes in different tissues before and after starvation. Note: E: eyestalk; M: muscle; HE: hepatopancreas; H: heart; G: gill; I: intestine. * means significant difference (*p* < 0.05).

To explore the relationship between DNMTs and changes in methylation levels in the tissues, the expression characteristics of *DNMT1* and *DNMT3b* genes in different tissues were analyzed with qRT-PCR (Figure 4). Under starvation stress, the expression levels of both DNMT1 and DNMT3b in the intestinal and hepatopancreatic tissues were significantly down-regulated (*p* < 0.05). The expression of both genes was also down-regulated in the eyestalk. In other tissues, the expression of DNMT1 and DNMT3b was significantly up-regulated, with DNMT3b up-regulation at a greater level of significance.

### 3.3. Spatial Distribution Analysis of mRNA Signals of DNMT1 and DNMT3b 

**Figure 5:**
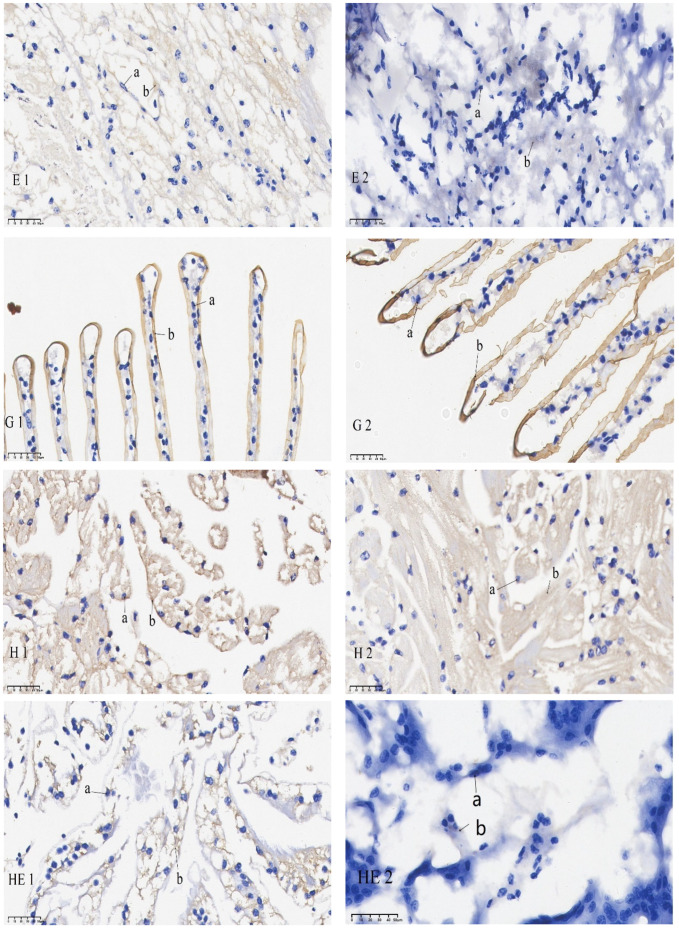

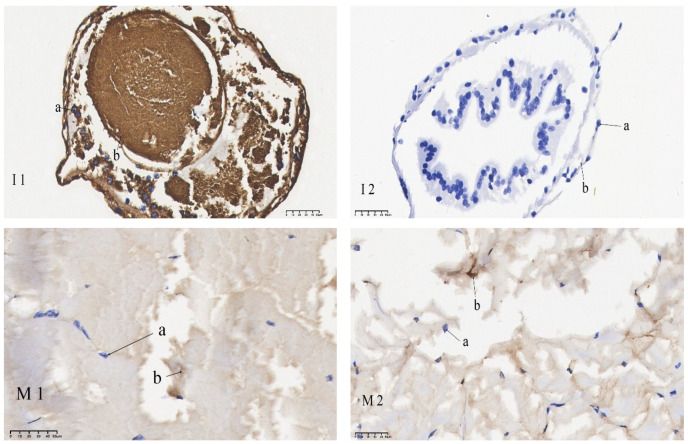
mRNA hybridization signal of *DNMT1* in different tissues of control and starvation groups. Note: E: eyestalk; M: muscle; HE: hepatopancreas; H: heart; G: gills; I: intestinal; 1: control group; 2: starvation group a: nucleus; b: positive signal.

The results of the in situ hybridization of DNMT1 are shown in Figure 5: in the eye stalk, DNMT1 was predominantly expressed in the inner medulla, outer medulla, and terminal medulla. In the gills, its expression was concentrated in the gill filament epithelium and stratum corneum cells. In the heart, it was chiefly detected in cardiomyocytes. In the hepatopancreas, it was expressed in the epithelial cells. In the intestines, it was mainly expressed in the inner and outer walls, basement membrane, and connective tissue. In the muscles, it was mainly localized in the sarcoplasm. Under starvation, mRNA signaling in the gut, hepatopancreas, and eye stalk was significantly reduced compared to the control group. No significant differences in mRNA signaling were found in the gills, muscles, and heart when comparing the pre-starvation group to the post-starvation group.

**Figure 6:**
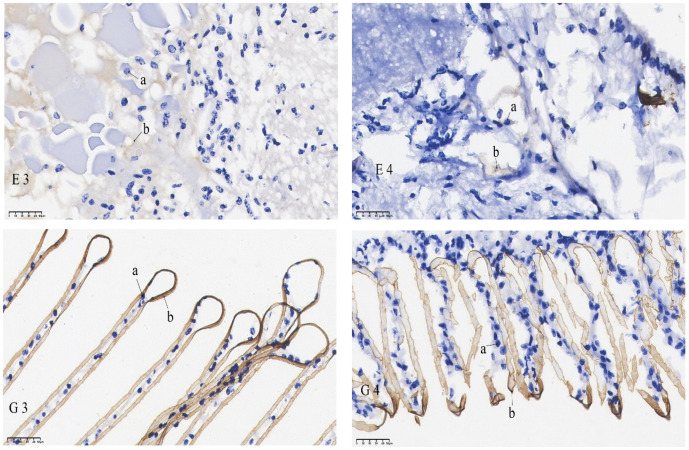

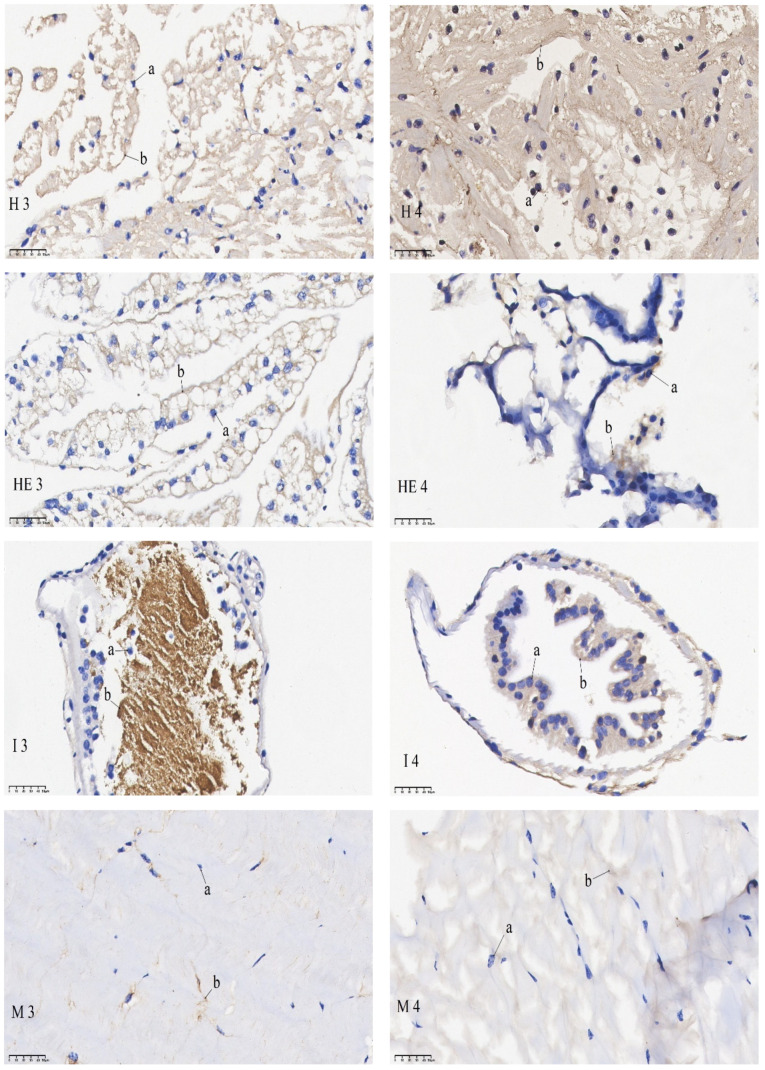
mRNA hybridization signal of *DNMT3b* in different tissues of control and starvation groups. Note: E: eyestalk; M: muscle; HE: hepatopancreas; H: heart; G: gills; I: intestinal; 3: control group; 4: starvation group; a: nucleus; b: positive signal.

The results of the in situ hybridization of DNMT3b are shown in Figure 6: DNMT3b was predominantly expressed in the outer medulla of the eye stalk and was also detectable in the retina. In the gills, its expression was concentrated in the gill filament epithelium and stratum corneum cells. In the heart, it was primarily found in cardiomyocytes. In the intestines, it was expressed in the basement membrane and, to some extent, in the intestinal lining. In the muscles, it was mainly localized in the sarcoplasm. The mRNA signals of the eye stalks, hepatopancreas, and intestines in the starvation group were significantly lower than those of the control group. No significant difference in the mRNA signal was found in the gills when comparing the pre-starvation group to the post-starvation group. For the muscles and heart, the mRNA signal was stronger in the starvation group than in the control group.

## 4. Discussion

### 4.1. Tissue-Specific Difference in mtDNA Methylation Levels and Potential Mechanisms

In previous studies, we found that some regions of the *COX3* and *ND2* genes in the mitochondrial genome of the muscle tissue of *E. carinicauda* were methylated under starvation stress [23,24]. Both genes are mainly responsible for regulating the shrimp’s energy metabolism. Mitochondrial genome methylation reduces the level of energy metabolism in tissues, and in the process, methylation regulates the expression of genes related to energy metabolism, such as *COX3* and *ND2* [25,26]. Nevertheless, the level of energy metabolism is not consistent among tissues in different organisms, and the level of the regulation of energy metabolism by mitochondrial genome methylation in different tissues is different under starvation stress levels. The results of this study demonstrate that mtDNA methylation levels of mitochondrial DNA across different tissues have distinct variations, with a ranking from highest to lowest as follows: intestine, hepatopancreas, gill, heart, muscle, and eyestalk. The variations can be reasonably correlated to the biological functions of these tissues in response to starvation. In contrast, the mtDNA methylation rate for all tissues in the control group was “0”, which is consistent with our previous findings. Starvation, an external environmental factor, is directly related to the regulation of energy metabolism. When an individual is not in a starvation state, the level of energy metabolism in tissues does not require the regulation of DNA methylation, and the expression of the *COX3* and *ND2* genes is likewise not regulated by DNA methylation, Therefore, the methylation sites on the 16# and 21# sequences on the *COX3* and *ND2* genes in this study were not methylated.

As we all know, the intestine is the primary tissue that responds to starvation stress, while other tissues like eyestalks are markedly less sensitive to such conditions. During starvation, the intestine and hepatopancreas, which serve as the main sites for nutrient exchange, absorption, and energy storage, need to reduce their energy metabolism, which is accomplished by a decrease in energy consumption while maintaining normal tissue functions. This may explain why the methylation levels of mtDNA in the intestine and hepatopancreas were high. Mitochondrial genome DNA methylation levels in the eye stalk were found to be significantly lower compared to other tissues. The main functions of the eye stalk in shrimp are related to vision, perception, endocrine functions, and maintaining body spatial position and equilibrium, etc. Since the eye stalk is less affected by hunger, the methylation level of mtDNA was found to be much lower compared to the other tissues. From another perspective, this illustrates the close relationship between the main biological function of mtDNA methylation and the regulation of energy metabolism.

### 4.2. The Regulatory Mechanisms of DNMT1 and DNMT3b on mtDNA Methylation in Different Tissues

DNMT1 and DNMT3b are important DNA methyltransferases, and earlier studies of the nuclear genome have clearly shown that the elevated expression of *DNMT1* and *DNMT3b* causes an increase in DNA methylation levels [27,28]. The results of this study show that most of the tissues in *E. carinicauda* also demonstrate a similar pattern. For example, under starvation, the expression of *DNMT1* and *DNMT3b* in gill tissue increased, and the level of mtDNA methylation in this tissue was also high. Conversely, in the eye stalk, where *DNMT1* and *DNMT3b* expression remained consistently lower than in the other tissues, the methylation levels of mtDNA were also much lower. The mtDNA methylation levels in muscle and heart were also found to be positively correlated with the changes in the expression of *DNMT1* and *DNMT3b*. These findings align with those of Binghua Liu et al., who reported that hypoxia stress in flounder, *Paralichthys olivaceus,* induced concomitant increases in nuclear DNA methylation levels and the expression of *DNMT1/DNMT3b* in gills [29], which implies a similar positive correlation mechanism in the tissues of *E. carinicauda*. Intriguingly, *DNMT1* and *DNMT3b* exhibit distinct regulatory effects on mtDNA methylation across different tissues. Our analysis revealed that, under starvation, the expression of *DNMT1* exhibited no significant differences across the gill, heart, and muscle, whereas the expression of *DNMT3b* was significantly elevated in these tissues. These results indicate that *DNMT3b* is likely the primary factor governing mitochondrial genome methylation in these three tissues. This phenomenon may be attributable to the de novo methylation function exerted by *DNMT3b* within these tissues. Other research findings have shown that, when the zebrafish, *Danio rerio*, is in a hypoxic condition, its gill shows a regenerative phenomenon. Meanwhile, the mitochondria-rich cells (MRCs) within the gill undergo cellular renewal and substitution [30,31], We speculate that when *E. carinicauda* is confronted with starvation stress, similar regeneration and replacement likely occurs in the cells of the gill. The in situ hybridization analysis of the gill tissue revealed that the nuclear density in the gill tissue in the post-starvation state was significantly higher than that in the pre-starvation state. This indicates that as cells undergo continuous differentiation, the expression level of de novo methylase *DNMT3b* also rises accordingly. As shown in a previous study, starvation compromises the basic muscle structure of red swamp crayfish, *Procambarus clarkii* [32]. The in situ hybridization results for the muscle of *E. carinicauda* confirm that damage occurs to the muscle during starvation. The heart is the main tissue for oxygen transportation and blood circulation, and in this study, the expression level of *DNMT3b* in the heart also increased after starvation. Thus, as *a de novo* methyltransferase, *DNMT3b* may be mainly involved in regulating mtDNA methylation in energy metabolism. Unexpectedly, in this study, the intestinal and hepatopancreatic tissues in the post-starvation group showed hypermethylation, but the expression levels of *DNMT1* and *DNMT3b* and the mRNA hybridization signal in these tissues were significantly reduced, which is contrary to the expected outcomes. We suggest the following explanations: When individuals are under starvation stress, mtDNA methylation levels in tissues increases to reduce energy metabolism. Because the intestine and hepatopancreas of shrimp are important tissues that are mainly responsible for energy digestion, absorption and metabolism in the body, during starvation, they may be under the greatest initial stress. This would trigger increases in the methylation levels in these tissues. The increased methylation levels will then inhibit the increased levels of gene expression in relation to the regulation of methyltransferase. Therefore, we observed that the expression levels of methylases in these two tissues were not high. The methyltransferases previously expressed in these two tissues would also be transported to other tissues simultaneously. Therefore, under these influences, we observed that the expression level of methyltransferase was relatively high in tissues other than the intestine and hepatopancreas. In addition, we observed that the level of methyltransferase was highest in cardiac tissue. This phenomenon could also be explained by the inference from above.

## 5. Conclusions

In this study, we used the bisulfite method (BSP) to detect the methylation of the mitochondrial genome in the intestine, hepatopancreas, gills, eyestalks, muscles, and heart between pre-starvation and post-starvation states. We also used in situ hybridization and qPCR techniques to analyze the expression of *DNMT1* and *DNMT3b* involved in methylation regulation in different tissues. The results showed that the methylation rate was highest in intestinal tissue, followed by the hepatopancreas, gills, heart, muscle, and eye stalk. Significantly different expression levels of *DNMT1* and *DNMT3b* were also found in the intestine and hepatopancreas between pre-starvation and post-starvation states, with higher expression levels occurring at pre-starvation, and lower expression levels post starvation. The expression levels of *DNMT1* and *DNMT3b* in heart and muscle increased after starvation, and the expression levels of *DNMT1* and *DNMT3b* in the eye stalk were low and decreased significantly after starvation. The in situ hybridization of *DNMT1* and *DNMT3b* further verified the results: the mRNA signal in the intestine and hepatopancreas from the starvation group was significantly weaker than that of the control group. No significant difference in the mRNA signal intensity was found in the gill, muscle, and heart of the starvation group compared to the control group. The mRNA signal in the eye stalk of the starvation group was weaker than that of the control group. These results, for the first time, confirm that differences in mtDNA methylation levels exist among different tissues of *E. carinicauda*, which may be closely related to their biological functions.

## Figures and Tables

**Table 1:** BSP-amplified primary sequence.

Name	Sequence
16#	TGGTTTTTTGATAGTGATTTTATATCGTTTATATATGTGT
	TATTTTTTAGTTAATTATTATTTTGGATTCGAAGTTGTTG
	TTTGGTATTGATATTTTGTCGACGTAGTTTGATTTTITTT
	TTATATTTCGTTTATTGATGAGGAGGATAATTTTATATTT
	ATTTAGTATAATTGTATAATTGATTTTTAATTAATAAGTT
	TGGTATTTTTAGGATAG
21#	AAGTGTTAGTTAATTTAGGTTTAAATCGAATCAATAAATA
	TACCCCCGCCGCTACTAAAGTAGAAGAATGCACTAAAGCT
	GATACAGGCGTAGGAGCTGCCATTGCAGCTGGTA

**Table 2:** Related primers used in this study.

Name	Sequence	Purpose
16#-F	TGGTTTTTTGATAGTGATTTTATAT	BSP primers
16#-R	CTATCCTAAAAATACCAAACTTATTATTA	
21#-F	AAGTGTTAGTTAATTTAGGTTTAAAT	
21#-R	TACCAACTACAATAACAACTCCTAC	
D1-qRT-F	CATCCAGACAAAAAGCAAGG	qRT primers
D1-qRT-R	CCAGTATGAGGCAAACACCA	
D3- qRT-F	GCCGGTAGGATCAAACAACC	
D3-qRT-R	CAACGAACCTGCCAAAAATG	
18S-F	TATACGCTAGTGGAGCTGGAA	
18S-R	GGGGAGGTAGTGACGAAAAT	
D1-F	AGCCATGACCCAGTCACGTAATGCCGGGAGTGTTATCCCA	Recombination primers
D1-R	CTTACTTCTGACAACGATCGCCAGTGAAGAGTGACAATTTTGGA	
D3-F	AGCCATGACCCAGTCACGTAATGATGGAATCAAACTATGAAGTACTTG	
D3-R	CTTACTTCTGACAACGATCGTTATCTGAATCTTTCTTGTCGTTTGG	
Pspt18-F	CGATCGTTGTCAGAAGTAAGTTGG	
Pspt18-R	TACGTGACTGGGTCATGGCTG	
TanD1-sp6	ATTTAGGTGACACTATAGAATACAAGCTTGCATGCCTGCGCAACAAATTCGTC	DIG probes
TanD3-sp6	ATTTAGGTGACACTATAGAATACAAGCTTGCATGCCTGCTCATCAAGCGTT	

**Table 3:** Methylation rates in different tissues.

Tissues	Total Methylation Rate of Control (%)	Numbers of 16&21# Methylation Sites	Numbers of Total Methylation Sites	Total Methylation Rate of Starvation (%)
Intestine	0	206	570	36.29 ± 2.16
Hepatopancreas	0	184	570	30.5 ± 1.46
Gill	0	151	570	26.4 ± 0.98
Heart	0	124	570	21.9 ± 0.83
Muscle	0	109	570	19.3 ± 0.76
Eyestalk	0	31	570	5.76 ± 0.78

## Data Availability

The authors declare that all data supporting the findings of this study are available from the corresponding authors upon reasonable request.
